# Unusual Configurations of Personality Traits Indicate Multiple Patterns of Their Coalescence

**DOI:** 10.3389/fpsyg.2018.00187

**Published:** 2018-02-20

**Authors:** Jüri Allik, Martina Hřebíčková, Anu Realo

**Affiliations:** ^1^Department of Psychology, University of Tartu, Tartu, Estonia; ^2^Estonian Academy of Sciences, Tallinn, Estonia; ^3^Institute of Psychology, Czech Academy of Sciences, Brno, Czechia; ^4^Department of Psychology, University of Warwick, Coventry, United Kingdom

**Keywords:** personality traits, five-factor model, trait configurations, NEO PI-R/3, personality mutants

## Abstract

It is widely accepted that the Five Factor Model (FFM) is a satisfactory description of the pattern of covariations among personality traits, which supposedly fits, more or less adequately, every individual. As an amendment to the FFM, we propose that the customary five-factor structure is only a near-universal, because it does not fit all individuals but only a large majority of them. Evidences reveal a small minority of participants who have an unusual configuration of personality traits, which is clearly recognizable, both in self- and observer-ratings. We identified three types of atypical configurations of personality traits, characterized mainly by a scatter of subscale scores within each of the FFM factors. How different configurations of personality traits are formed, persist, and function needs further investigation.

## Introduction

Although sporadically criticized, the Five Factor Model (FFM) is a nearly universally accepted approach to explaining how individuals typically describe their own or somebody else's personality (McCrae and John, 1992; Goldberg, 1993; John et al., 2008; McCrae and Costa, 2013). Louis Thurstone invented the method of factorial analysis (Thurstone, 1931), which, according to his belief, can be used as a tool to simplify the complexities of social and psychological phenomena into a limited number of elements (Thurstone, 1934). However, it was the next generation of researchers who noticed that there is only a limited number of independent dimensions underlying the vast array of personality adjectives in existence across languages, which are, in turn, reflected in the large number of items in personality questionnaires (Fiske, 1949; Norman, 1963; Digman, 1989; Goldberg, 1993). Even if the exact number of underlying dimensions is still debated, most researchers seem to agree that the number of independent personality factors is relatively small and not very much higher than five, which was also Thurstone's original hunch (Thurstone, 1934).

When people describe their own or somebody else's personality, many of the descriptors typically go hand-in-hand with each other. For instance, people who are often angry also experience many other negative emotions. Similarly, individuals who have vivid imaginations are regularly more open to trying new food and entertaining new ideas and values. And those who are motivated to achieve are usually also self-disciplined and methodical. It was exactly the analysis of these covariations between personality adjectives—Allport and Odbert (1936) identified thousands of trait names in the Webster's dictionary—and questionnaire items which led to the discovery of the FFM, according to which, there are five main personality factors or dimensions, usually labeled Neuroticism, Extraversion, Openness to Experience, Agreeableness, and Conscientiousness (McCrae and Costa, 1987; John et al., 2008). Paul T. Costa, Jr. and Robert R. McCrae developed perhaps one of the most versatile instruments for the measurement of the FFM, the Revised NEO Personality Inventory (NEO PI-R; Costa and McCrae, 1992), which was specifically designed to measure 30 distinctive personality traits grouped into the abovementioned Big Five dimensions. Now, 25 years later, the NEO PI-R and its latest version, the NEO PI-3, have been translated into at least 37 different languages and used in more than 60 different countries or cultures (Allik et al., 2017).

As the pattern of covariation between these 30 traits was shown to be fairly uniform across different languages and cultures, there was good reason to suggest that these five factors of personality constituted a human universal (McCrae and Costa, 1997). It is indeed surprising that relatively few problems occurred in the replication of the original NEO PI-R/3 factor structure in other languages and cultures (e.g., Rolland, 2002). Issues have arisen, nevertheless; the E5: Excitement Seeking scale, for example, has a tendency to load strongly on the Openness, not only the expected Extraversion, factor in many African countries (Zecca et al., 2013). Another subscale which has demonstrated some inconsistency in various applications is N5: Impulsiveness. Providing an example of how to interpret personality profiles, the authors of the NEO PI-R manual presented a personality profile of a 32-year-old married woman (Case A, Figure 2), which was characterized by a deviation of N5 from other Neuroticism subscales: “Note that there is a considerable scatter within the N domain. This women considers herself to be very high in N1: Anxiety and high in N3: Depression and N6: Vulnerability, but very low in N5: Impulsiveness—perhaps as a result of her high level of Conscientiousness” (Costa and McCrae, 1992, p. 19). Although devised as a marker of Neuroticism, N5: Impulsiveness has also a tendency, in some cultures, to carry a meaning associated with Extraversion or Conscientiousness (Konstabel et al., 2002). Although a general pattern of covariation defining the FFM is reasonably stable across cultures and samples, small individual variations may still emerge in the networks to which the subscales appear to belong. These relatively small “anomalies” in factor structure may be caused by statistical flukes, but also by more systematic factors which affect all participants in the sample similarly, including culture (Konstabel et al., 2002).

Even after proposing parallel analysis and other advanced techniques for deciding how many factors to retain (Horn, 1965; Reise et al., 2000; Ledesma and Valero-Mora, 2007), intuition and experience still play an important role in the determination of the number-of-factors problem in principal component and exploratory factor analysis. Because there are no strict rules, the exact number of independent personality factors is still debated by personality psychologists (Block, 1995, 2010). Some researchers maintain that the right number is less than five (Eysenck, 1991, 1992); others believe that the FFM ignores an additional sixth dimension (Ashton et al., 2006; Ashton and Lee, 2010). In any case, proposing a different number of factors means that the same pattern of covariation may have different interpretations. However, one possibility is that the number of personality dimensions is not a constant but a variable, the value of which can therefore change. For example, it was noticed that the FFM could also exist in truncated form. If somebody, such as a politician, for instance, is not known personally but mainly through media, people may describe his or her personality using only two or three factors instead of all five (Caprara et al., 1997, 2003). Another interesting observation is that the full version of the FFM may only emerge in sufficiently complex societies. It is possible that, in some small-scale traditional societies, a smaller number of FFM personality dimensions is enough to describe people's personality traits. For example, the forager-horticulturalist Tsimane people of Bolivia do not seem to have the customary five-factor structure of personality but only the Big Two (Gurven et al., 2013, 2014). Analogously, Toomela (2003) found that individuals who primarily used everyday concepts in their thinking did not reveal the usual five-factor personality structure, but rather a simpler version of only two or three factors. Although most deviations from the FFM in Toomela's (2003) data can be ameliorated (Allik and McCrae, 2004), they nevertheless demonstrate that the number of extracted factors may be not rigidly fixed; the optimal number of factors may change as well. However, in all these cases we are talking about a reduced version of the well-known personality structure, not about previously unknown factors.

Although prototypic five factors are recoverable from almost every data collected so far, it is unlikely that we are dealing here with a human universal in the strict sense. Universal means that something is characteristic to all members of a class, without limits or exceptions. Obviously, such a requirement is too strong because we can imagine that the FFM does not fit every human being but only most of them (Allik et al., 2013; Allik and Realo, 2017). Thus, we are talking about a near-universal for which it is necessary to establish yet for how many individuals the FFM is the best possible description of their personality.

This presents us, therefore, with a challenge. Personality psychologists have not, so far, been able to agree on how to measure the so-called personal fit of various latent trait models, including the FFM. There is no universally accepted method of how to compute an individual person-fit to the FFM, or any other latent trait personality model. Let us remember that all latent trait models use a matrix of covariations that is initially computed on the basis of interindividual differences among a group of participants. Two traits are correlated when individuals have on average a similar ranking on those two traits. Two traits are orthogonal when, based on individual rankings on one of the traits, it is impossible to predict anything certain about rankings on the other trait (Allik et al., 2015).

Because individual ranking is specified relative to a group it is believed that the pattern of covariations between personality traits provides very little information about the individuals who are involved in these rankings (Borsboom, 2005). However, this is not necessarily true because an individual, for example, with a very low score on a certain trait are unlikely to occupy a very high position in the ranking of individuals on that trait. It became popular to believe that the only way to get information about the covariation between personality traits within an individual is to administer the same personality questionnaire repeatedly over a relatively short period of time (Borkenau and Ostendorf, 1998; Borsboom et al., 2003; Molenaar et al., 2003). Because the intraindividual pattern of covariation may not coincide with the interindividual pattern, it is believed that standard personality models such as the FFM, which can be derived only from the group data, do not apply to some, or even any, individuals in that group (Borsboom et al., 2003; Molenaar and Campbell, 2009).

However, assessing the fit of measurement models at the individual level does not necessarily involve the repeated administration of the same questions. For example, a number of person-fit statistics (e.g., the caution index, the norm conformity index, and the individual consistency index) that have been developed in educational and applied psychology settings measure how well statistical models fit at the level of the individual (Meijer and Sijtsma, 2001; Karabatsos, 2003; Albers et al., 2016). Most person-fit statistics used in testing mental abilities are based on comparing the individual response pattern with a general response pattern of the group. This approach, however, is not well adapted for multifactor models, because it is not a good idea to measure deviations from an average response pattern. Even a personality profile that deviates substantially from the group average could be regarded as normal, because nothing in theory or practice prevents this individual from having these particular levels of traits. For example, no extravert or introvert can be regarded as deviants from the norm only because the average person scores happen to be between these two extremes.

One elegant solution for the person-fit problem applied to latent-trait models came from Reise and Widaman (1999), who proposed that an individual's contribution can be calculated with the chi-square (χ^2^) statistic, which measures the log likelihood that the observed covariance matrix is reproduced by the statistical model. To this end, they partitioned the model's overall χ^2^ value into the subjects' individual contributions (by observing changes that resulted after one by one elimination of participants from the sample) such that a relatively large decrease in fit would result if an individual who contributes more to the overall model fit and more to the increase in χ^2^ value. Unfortunately, this proposal inspired only very few followers. One possible reason is insensitivity of this method to the number of the latent variables and consequently to FFM or any other particular version of the model (Allik et al., 2012).

Another approach to the person-fit of the FFM may be based on the observation that all personality inventories were modeled on Campbell and Fiske's basic idea about convergent and discriminant validity (Campbell and Fiske, 1959). The main proposal of their seminal paper seems, nowadays, self-evident: measures of the same general trait made by different subscales should agree (converge) better than measures of different traits. As a matter of fact, this simple idea is behind every omnibus personality questionnaire that is currently used. Indeed, it is expected, for example, that Neuroticism subscales have more similar scores that discriminate them from the scores that Extraversion subscales have (Allik et al., 2012). In other words, it assumes that the same individuals have approximately the same scores and consequently similar ranking on subscales or facets measuring the same trait. It is easy to see that *Intraclass Correlation* (*ICC*) is then exactly the measure that shows how well the FFM (or any other latent trait model) fits an individual. For instance, we can split the total variance produced by the 30 NEO PI-R/3 individual facet scores into two components: the within-factor (σ^2^_*W*_) and the between-factor (σ^2^_*F*_) variance. The *ICC* is defined as the ratio between the variance attributable to the mean differences between factor variance and the total variance: *ICC* = σ^2^_*F*_/(σ^2^_*W*_ + σ^2^_*F*_). The *ICC* in fact detects individual patterns of response in which the scores of the subscales measuring the same factor are maximally similar but with mean levels that differ substantially.

Based on this definition of the person-fit, it is also easy to formulate the most conspicuous feature of an anomalous personality profile. This is a large scatter of the subscale scores within the same dimension or factor. This is exactly a reason why the within-factor variance (σ^2^_*W*_) can become relatively large compared to the between-factor variance (σ^2^_*F*_). For example, if one or several subscales have high scores but some other subscales of the same dimension have low scores, then it is a sign that we probably have a deviation from the canonical FFM (Allik et al., 2012). Indeed, imagine that we have a participant who scored considerably below the average level of the O3: Feelings subscale, which according to the NEO PI-R's manual indicates that this person has somewhat blunted affects and does not believe that feeling states are much of importance (Costa and McCrae, 1992). Because the subscales of the same trait are expected to be correlated, we cannot presume that the same individual will score very high on O6: Values showing that this individual is above average flexible to reexamine her or his social, political, and religious values. Hundreds of studies have shown that social conservatism and tampered emotional life usually go hand in hand. If we find an individual for whom this association is missing or even reversed then it should be viewed as a deviation from the common pattern characterizing the FFM.

When *ICC* was computed for individual NEO PI-R/3 scores of participants from Belgium, the Czech Republic, Estonia, and Germany, it turned out that the absolute majority of participants from these four countries had a good fit to the FFM, based on their *ICC* or person-fit values (Allik et al., 2012). It was also demonstrated that, if the FFM explains a considerable proportion of variance, then it is inevitable that a majority of participants are required to have sufficiently high individual *ICC* or person-fit scores (Allik et al., 2012). Nevertheless, a small group of participants in each of these four countries had unusual personality profiles, which did not fit the FFM, but, at the same time, agreed with the opinion of the acquaintances or relatives who judged these individuals. Thus, people who know the individuals in this group of atypical respondents relatively well recognize the anomalous constellations of personality traits. In this study, we provided two examples of these anomalous personality profiles (Allik et al., 2012; Figure 4B and 4C). One of these cases (4B) was a Flemish introvert who nevertheless appeared to be an above average warm, affectionate, and friendly person. She was also, according to her own and acquaintances' opinion, a prosaic person, who preferred to keep her mind on the task at hand. However, she scored high on some other facets of Openness, having, for example, a readiness to reexamine her social, political, or religious values. According to the customary FFM, one cannot be high on one indicator of Openness but very low on various others. Another example (4C), a 33-years old Czech women, was highly neurotic, as judged by her score on N1: Anxiety, but she had no feelings of embarrassment or inferiority, which are at the core of another facet of Neuroticism, N4: Self-Consciousness. Again, a basic idea for the FFM that anxiety and feelings of inferiority most likely coexist was violated (Allik et al., 2012).

Although the number of these deviations from the canonical pattern—we also can call them “personality mutants”—seems to be small, their existence may require a revision of one of the basic assumptions of personality theory. According to this assumption, there is only one possible way in which basic elements—personality facets—can be coalesced into higher order factors. It seems that the FFM assumes, tacitly at least, that there is only one configuration into which traits measured on the level of subscales can be grouped. The authors of the NEO PI-R Manual obviously recognized that some individual personality profiles may deviate from a prototypical FFM but they did not formulate any criteria how to recognize these deviants (Costa and McCrae, 1992). These deviations were not regarded as systematic but rather due to measurement errors or statistical accidents. However, nothing in the FFM, that we know of, would prevent us from supposing that there are alternative scenarios in which lower order traits or facets can merge into higher order factors or dimensions. As we already talked above, N5: Impulsiveness—inability to control own cravings and urges—is a faithful indicator of Neuroticism, along with other facets, such as N1: Anxiety, N2: Angry Hostility, N3: Depression, N4: Self-Consciousness, and N6: Vulnerability (Costa and McCrae, 1992). However, a minority group may exist among neurotics who have no difficulty resisting temptations and who a have high tolerance for frustration. On the contrary, they may be deliberate and cautious in avoiding impulsive actions. Although impulsive behavior characterizes Neuroticism, it may fall out of the ensemble and become an indicator of some other factor, such as Conscientiousness (Costa and McCrae, 1992).

It is important to notice that the FFM remains largely cryptic about the way in which facets are coalesced into factors (Costa and McCrae, 1995, 2017). As McCrae and Costa (2017) write: “The structure of personality at the level of facets appears to be ill-defined; some system of facets is needed and useful, but which system is chosen is to some extent arbitrary” (Costa and McCrae, 2017, p. 18). A recently proposed network approach seems to provide more concrete predictions about how facets are amalgamated into different factors (Cramer et al., 2012). According to this approach, coherent factors are formed based on networks of mutual dependencies between different, more specific life events that may variously have causal, homeostatic, or logical sources. Bidirectional dependencies will form if two components influence one another (and, as such, create a feedback loop): for example, after a sleepless night worrying, one may feel stressed out and tired the next day, as a result of which, one may not sleep the following night either because of worries about yet another sleepless night (Cramer et al., 2012). However, at a formal level, these two approaches—the more traditional FFM and the network approach—may not be as distinctive as they first appear (Asendorpf, 2012; Wright, 2017); there is a general need for combinatory rules about how various trait coalitions are formed, even if some of them are not very frequent. It seems that the FFM requires an amendment by supplementing our understanding of how facets are coalesced into more general factors, and how rare trait configuration are occasionally formed, with some additional elaboration. Although some authors think that six, not five factors provide a superior description of the personality structure (Ashton et al., 2006, 2014), the same problem of deviations from the canonical personality structure remains for any other number of factors.

In this study, we take the first steps toward describing and understanding how atypical personality trait groupings may emerge in a normal population. For the first time, we attempt to describe individuals with unusual personality profiles, which deviate from the conventional FFM.

## Methods

### Czech sample

The Czech sample included 808 individuals (329 males, 479 females) who were recruited in a series of previous studies (McCrae et al., 2004; Allik et al., 2012). They ranged in age from 14 to 83 years, with a mean of 35.7 (*SD* = 14.2) years. Peer ratings were provided by 909 raters (377 males, 532 females) aged 14–83 years (*M* = 35.8; *SD* = 14.3) who participated in one of two research designs. In the self-other agreement studies (*N* = 616), each participant provided a self-report and was rated by one informant. In the consensus study, 196 participants (85 males and 111 females aged 17–77 years; mean age 36.4, *SD* = 15.2) provided a self-report and were each rated by three informants. All participants used the Czech version of the NEO PI-R questionnaire (Hrebíčková, 2002).

### Estonian sample

Data used in this study were reported in several previous publications (e.g., Allik et al., 2016; de Vries et al., 2016). Participants came from the Estonian Biobank cohort whose data were collected by the Estonian Genome Centre (EGC) of the University of Tartu (Leitsalu et al., 2015). Participants were recruited on a voluntary basis from the Estonian resident adult population (aged over 18 years). A small fraction of Estonian Biobank participants was also asked to complete a personality questionnaire. After removing 245 participants with incomplete or missing data (e.g., no observer-reports were available), there were 3,345 participants (1,984 women and 1,361 men) with a mean age of 46.4 years (*SD* = 17.0, ranging from 18 to 91 years) who completed the self-report form of the Estonian version of the NEO Personality Inventory-3 (NEO PI-3; McCrae et al., 2005), which is a slightly modified version of the NEO PI-R (Kallasmaa et al., 2000). All these participants nominated somebody who knew them well, and these informants were asked to rate the personality of the participant using the other-report version of the Estonian NEO PI-3. Of the informants, 2,331 were female (71.1%) and 948 were male (66 did not report their gender). The mean age of informants was 41.8 (*SD* = 15.9) years. The informant questionnaire contained several questions about the relationship to the target, asking, for example, how long they knew the individual. The mean duration of acquaintance was 23.2 years (*SD* = 15.1).

Both the Czech and Estonian versions of the NEO PI-R/3 have 240 items that measure 30 personality facets, which are grouped into the five FFM domains—Neuroticism (N), Extraversion (E), Openness to Experience (O), Agreeableness (A), and Conscientiousness (C)—such that each domain score is a composite of six facet scores. The items are answered on a five-point scale (0 = false/strongly disagree −4 = true/strongly agree). Data (in Excel format) can be downloaded from the following link: https://osf.io/u4hyk.

## Results

To start, we converted mean raw scores of the 30 NEO PI-R/3 facets into *T-*scores (mean equal to 50 and standard deviation equal to 10) using separate mean and standard deviation values for males and females in two separate age categories (i.e., either younger or older than 30 years). This standard conversion was done separately for self- and other-ratings (Costa and McCrae, 1992). Because of the conversion, we obtained *distinctive* scores characterizing how much each individual scored lower or higher on each trait relative to his or her age group. Therefore, the distinctive *T-*scores were separated from the *normative* scores, which characterize how much an individual is similar to other individuals, on average.

Next, we used a principal component analysis to extract five factors from all four *T-*scored datasets—self- and other-reports for the Czech and Estonian participants. Because even good multifactor personality instruments hardly ever fit their theoretically intended factor structure—maximally large loadings on the intended and a near zero loading on unintended factors—it is necessary to assume that some cross loadings have substantially non-zero values (Marsh et al., 2014). One possibility is to use the original American Normative Structure (Costa and McCrae, 1992; Table 5) as a benchmark against which any extracted NEO PI-R/3 factor structure can be compared. This makes sense because the American version of NEO PI-R/3 is a prototype for all adaptations. In order to evaluate how congruent these four factor structures were with the original American Normative Structure, we rotated them toward this normative structure using a Procrustes rotation technique (McCrae et al., 1996). Table 1 demonstrates the factor loadings for these rotated structures and their congruence coefficients with the normative structure. Because the overall congruence coefficients were higher than 0.97 in all four datasets, we can safely say that all four extracted factor structures were nearly identical to the American Normative Structure (McCrae et al., 1996). The five extracted factors explained 59.2 and 63.5% of the total variance in the 30 by 30 matrix of covariations for Czech self- and informant-ratings, respectively. In the Estonian data, the percentage of explained variance for self- and informant-ratings was 60.0 and 64.5%, respectively.

**Table 1:** Factor loadings and congruences for Czech and Estonian self- and other-rated personality structures Procrustes rotated to the Normative American Structure (Costa and McCrae, 1992).

	**Self-ratings**						**Other-ratings**					
**NEO PI-R facet scale**	**N**	**E**	**O**	**A**	**C**	**Congr**	**N**	**E**	**O**	**A**	**C**	**Congr**
**CZECH DATA**												
N1:Anxiety	**0.83**	−0.11	−0.00	−0.04	−0.04	*0.98*	**0.83**	−0.09	−0.02	0.02	0.11	*0.96*
N2:Angry Hostility	**0.74**	0.03	−0.10	−0.40	−0.05	*0.98*	**0.65**	−0.01	−0.14	−0.53	−0.04	*0.98*
N3:Depression	**0.81**	−0.21	−0.01	0.01	−0.20	*0.99*	**0.80**	−0.19	0.04	0.09	−0.17	*0.98*
N4:Self-Consciousness	**0.70**	−0.23	−0.19	0.11	−0.13	*0.98*	**0.68**	−0.32	−0.13	0.15	−0.01	*0.95*
N5:Impulsiveness	**0.40**	0.44	0.20	−0.29	−0.37	*0.95*	**0.38**	0.47	0.15	−0.34	−0.34	*0.95*
N6:Vulnerability	**0.77**	−0.15	−0.04	0.04	−0.32	*0.99*	**0.76**	−0.09	−0.08	0.00	−0.35	*0.99*
E1:Warmth	−0.11	**0.73**	0.14	0.33	0.17	*0.99*	−0.13	**0.72**	0.16	0.41	0.11	*1.00*
E2:Gregariousness	−0.14	**0.73**	−0.09	−0.05	−0.04	*0.97*	−0.16	**0.68**	−0.01	−0.14	−0.12	*0.95*
E3:Assertiveness	−0.35	**0.51**	0.14	−0.40	0.31	*0.99*	−0.34	**0.50**	0.13	−0.45	0.32	*0.98*
E4:Activity	−0.09	**0.49**	0.13	−0.20	0.34	*0.98*	−0.04	**0.47**	0.05	−0.18	0.42	*0.98*
E5:Excitement Seeking	−0.03	**0.56**	0.17	−0.31	−0.15	*0.98*	−0.01	**0.56**	0.24	−0.36	−0.23	*0.96*
E6:Positive Emotions	−0.26	**0.67**	0.24	0.05	0.03	*0.95*	−0.25	**0.70**	0.24	0.08	−0.02	*0.95*
O1:Fantasy	0.23	0.19	**0.66**	−0.07	−0.19	*0.98*	0.18	0.16	**0.70**	0.02	−0.34	*0.97*
O2:Aesthetics	0.19	0.02	**0.76**	0.14	0.05	*0.99*	0.22	0.12	**0.74**	0.11	0.08	*0.98*
O3:Feelings	0.34	0.40	**0.61**	0.00	0.05	*0.99*	0.32	0.43	**0.59**	0.11	0.09	*0.98*
O4:Actions	−0.18	0.19	**0.57**	−0.03	−0.16	*0.98*	−0.13	0.20	**0.63**	−0.03	−0.09	*0.99*
O5:Ideas	−0.02	0.03	**0.81**	−0.03	0.14	*0.98*	−0.07	−0.05	**0.78**	−0.01	0.23	*0.98*
O6:Values	−0.22	0.15	**0.56**	0.10	−0.16	*0.95*	−0.22	0.14	**0.54**	0.30	−0.05	*0.80*
A1:Trust	−0.24	0.29	0.22	**0.52**	−0.06	*0.97*	−0.17	0.40	0.10	**0.56**	−0.10	*0.92*
A2:Straightforwardness	0.04	−0.09	−0.03	**0.72**	0.12	*0.97*	0.00	−0.07	0.01	**0.75**	0.20	*0.98*
A3:Altruism	−0.04	0.41	0.04	**0.62**	0.29	*0.98*	−0.07	0.34	0.08	**0.74**	0.26	*0.94*
A4:Compliance	−0.27	−0.09	0.04	**0.71**	−0.03	*0.99*	−0.28	−0.07	0.08	**0.77**	0.04	*0.99*
A5:Modesty	0.21	−0.20	−0.34	**0.51**	−0.10	*0.96*	0.17	−0.19	−0.15	**0.68**	0.06	*0.97*
A6:Tender-Mindedness	0.19	0.15	0.17	**0.56**	−0.12	*0.94*	0.13	0.19	0.17	**0.66**	0.06	*0.98*
C1:Competence	−0.42	0.21	0.11	−0.11	**0.64**	*0.98*	−0.37	0.09	0.15	−0.01	**0.74**	*0.99*
C2:Order	0.03	−0.12	−0.16	−0.03	**0.64**	*0.96*	0.10	−0.08	−0.12	0.08	**0.77**	*0.96*
C3:Dutifulness	−0.02	−0.02	−0.14	0.29	**0.78**	*0.95*	0.01	0.00	−0.10	0.36	**0.80**	*0.95*
C4:Achievement Striving	−0.07	0.25	0.14	−0.19	**0.75**	*1.00*	−0.10	0.18	0.13	−0.16	**0.78**	*1.00*
C5:Self-Discipline	−0.27	0.00	−0.12	0.04	**0.79**	*0.97*	−0.16	−0.01	−0.10	0.11	**0.83**	*0.95*
C6:Deliberation	−0.27	−0.32	−0.03	0.23	**0.58**	*1.00*	−0.32	−0.31	0.01	0.24	**0.64**	*0.99*
*Congruence*	*0.97*	*0.97*	*0.97*	*0.98*	*0.98*	*0.98*	*0.97*	*0.97*	*0.97*	*0.96*	*0.97*	*0.97*
	**Self-ratings**						**Other-ratings**					
**NEO PI-3 facet scale**	**N**	**E**	**O**	**A**	**C**	**Congr**	**N**	**E**	**O**	**A**	**C**	**Congr**
**ESTONIAN DATA**												
N1:Anxiety	**0.83**	−0.06	−0.08	−0.07	−0.07	*0.99*	**0.83**	−0.06	−0.10	−0.05	−0.04	*0.99*
N2:Angry Hostility	**0.73**	0.07	−0.14	−0.41	−0.08	*0.97*	**0.69**	0.04	−0.10	−0.52	−0.11	*0.99*
N3:Depression	**0.75**	−0.22	−0.09	0.04	−0.21	*0.97*	**0.75**	−0.23	−0.02	0.09	−0.20	*0.97*
N4:Self-Consciousness	**0.67**	−0.38	−0.09	0.01	−0.18	*0.96*	**0.68**	−0.41	−0.12	−0.01	−0.13	*0.95*
N5:Impulsiveness	**0.57**	0.27	0.08	−0.33	−0.30	*0.98*	**0.56**	0.28	0.00	−0.39	−0.33	*0.97*
N6:Vulnerability	**0.66**	−0.12	−0.17	0.02	−0.46	*0.99*	**0.68**	−0.08	−0.16	−0.11	−0.51	*0.97*
E1:Warmth	−0.22	**0.76**	0.08	0.25	0.16	*0.97*	−0.23	**0.75**	0.16	0.28	0.11	*0.98*
E2:Gregariousness	−0.21	**0.74**	0.03	−0.04	−0.02	*0.99*	−0.22	**0.76**	0.07	−0.07	−0.10	*0.98*
E3:Assertiveness	−0.29	**0.51**	0.18	−0.43	0.25	*0.98*	−0.26	**0.50**	0.16	−0.49	0.30	*0.97*
E4:Activity	−0.18	**0.63**	0.17	−0.29	0.32	*0.95*	−0.18	**0.63**	0.19	−0.28	0.34	*0.95*
E5:Excitement Seeking	−0.06	**0.58**	0.33	−0.31	−0.01	*0.94*	−0.14	**0.57**	0.39	−0.29	−0.08	*0.91*
E6:Positive Emotions	−0.20	**0.67**	0.31	−0.01	0.11	*0.95*	−0.22	**0.67**	0.33	0.08	0.14	*0.95*
O1:Fantasy	0.14	0.19	**0.70**	−0.10	−0.16	*0.97*	0.21	0.20	**0.69**	−0.01	−0.22	*0.97*
O2:Aesthetics	0.13	0.11	**0.65**	0.18	0.13	*0.99*	0.17	0.07	**0.72**	0.12	0.12	*1.00*
O3:Feelings	0.26	0.43	**0.57**	0.02	0.20	*0.98*	0.33	0.46	**0.50**	0.07	0.23	*0.98*
O4:Actions	−0.34	0.26	**0.58**	−0.12	−0.06	*0.96*	−0.36	0.36	**0.54**	−0.06	0.01	*0.95*
O5:Ideas	−0.12	0.11	**0.78**	−0.09	0.16	*0.99*	−0.15	0.06	**0.78**	−0.06	0.26	*0.99*
O6:Values	−0.30	−0.00	**0.57**	−0.08	−0.22	*0.97*	−0.35	0.12	**0.43**	0.18	−0.16	*0.83*
A1:Trust	−0.29	0.24	0.26	**0.49**	−0.10	*0.96*	−0.25	0.36	0.10	**0.62**	−0.04	*0.97*
A2:Straightforwardness	−0.08	−0.25	−0.01	**0.65**	0.11	*0.96*	−0.14	−0.15	−0.04	**0.69**	0.18	*0.98*
A3:Altruism	−0.08	0.38	0.03	**0.66**	0.16	*0.96*	−0.10	0.35	0.06	**0.75**	0.16	*0.93*
A4:Compliance	−0.15	−0.12	−0.07	**0.73**	−0.05	*0.99*	−0.29	−0.09	0.00	**0.76**	−0.02	*0.99*
A5:Modesty	0.14	−0.29	−0.24	**0.56**	−0.01	*0.96*	−0.01	−0.22	−0.06	**0.72**	0.12	*0.89*
A6:Tender-Mindedness	0.33	0.25	−0.06	**0.56**	0.14	*0.86*	0.23	0.28	0.10	**0.61**	0.15	*0.94*
C1:Competence	−0.46	0.16	0.08	−0.07	**0.67**	*0.99*	−0.46	0.09	0.12	0.02	**0.73**	*0.99*
C2:Order	−0.00	−0.00	−0.05	−0.02	**0.74**	*0.98*	0.00	−0.01	−0.07	0.04	**0.73**	*0.98*
C3:Dutifulness	−0.02	−0.03	−0.06	0.29	**0.74**	*0.97*	−0.07	−0.01	−0.03	0.37	**0.77**	*0.98*
C4:Achievement Striving	−0.03	0.25	0.16	−0.12	**0.73**	*1.00*	−0.11	0.22	0.19	−0.12	**0.76**	*1.00*
C5:Self-Discipline	−0.25	0.04	−0.05	−0.00	**0.78**	*0.98*	−0.24	0.05	−0.04	0.08	**0.81**	*0.98*
C6:Deliberation	−0.25	−0.27	−0.09	0.28	**0.60**	*1.00*	−0.27	−0.26	−0.04	0.26	**0.68**	*1.00*
*Congruence*	*0.96*	*0.97*	*0.96*	*0.98*	*0.98*	*0.97*	*0.96*	*0.97*	*0.96*	*0.96*	*0.98*	*0.97*

*Congr, congruence; N, Neuroticism; E, Extraversion; O, Openness to Experience; A, Agreeableness; C, Conscientiousness. Loadings higher than |0.40| on the intended factor are shown in bold and on the “wrong” factor in red. The Tucker's coefficients of congruence are shown in italics*.

**Figure 1:**
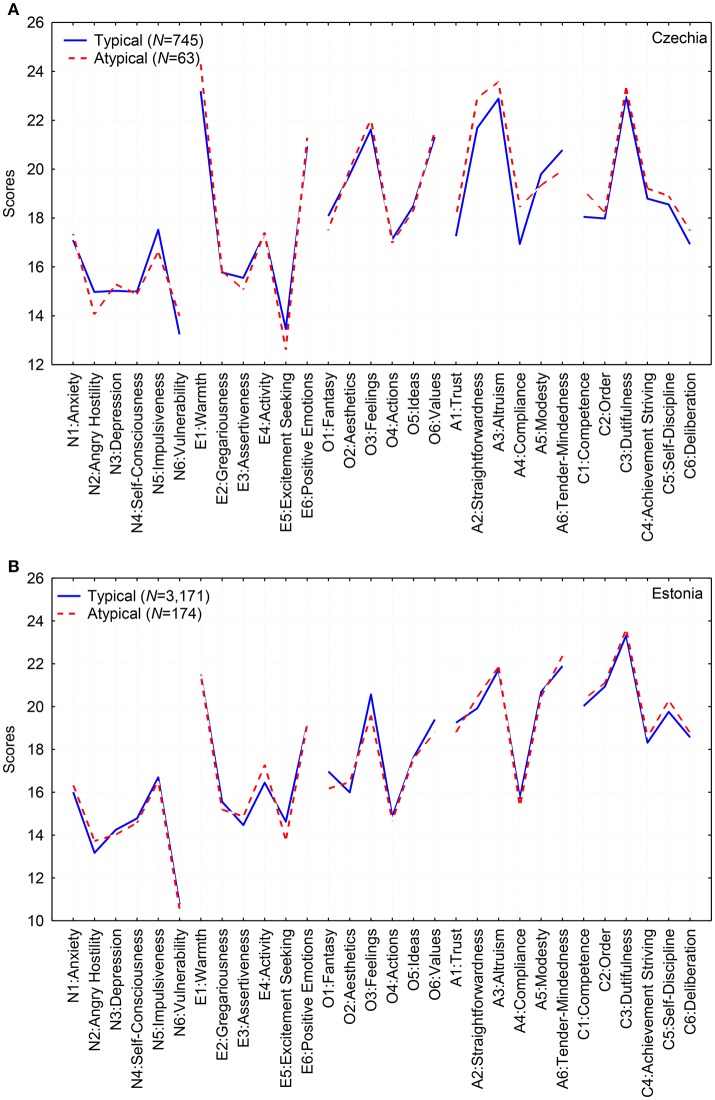
Mean profiles for participants with typical (solid blue lines) and atypical configurations (dotted red lines) of FFM personality traits in the Czech **(A)** and the Estonian **(B)** data.

From one of our previous studies, we know that a percentage of explained variance of around 60% approximately corresponds to the *ICC* mean value of around 0.40 (Allik et al., 2012; Figure 1A). Indeed, in the Czech data, the median *ICC* was 0.36 for self-ratings and *ICC* 0.39 for other-ratings. For the Estonian data, the median *ICC* was 0.42 and *ICC* = 0.47, respectively, for self- and other-ratings. The mean squares for the factors (i.e., between-factor variance) must be at least 2.76 times larger than the mean squares for the error (i.e., within-factor variance) with 4 and 25 degrees of freedom to exceed the critical value at a significance level of *p* < 0.05 (Allik et al., 2012). An *F*-ratio of 2.76 corresponds to a critical *ICC* value of 0.26. In the Czech sample, the *ICC* values were statistically significant at *p* < 0.05 for 67.6% of self-ratings and for 69.4% of other-ratings. In the Estonian sample, the percentage of *ICC* values that were statistically significant was 75.0 and 81.8% for self- and informant-ratings, respectively.

Thus, around 70–80% of the NEO PI-R/3 profiles are in statistically significant agreement with the FFM. These percentages of statistically significant *ICC* values explain why five factors are able to account for about 60% of the total variance. However, there is still about 40% of variance in the matrices of covariations that is due to measurement error, biases, and individual variation.

Before we compute self-other agreement, it is necessary to recognize that there are two principal ways of computing correlations between judgments of multiple informants. The trait agreement (*r*_T_) is computed separately for each of *K* personality traits across all *N* pairs of judges, while the profile agreement (*r*_P_) is calculated across *K* personality traits for each individual target-judge pair (Allik et al., 2015). Although these two forms of agreement are often believed to provide different information, they are in fact identical if data are both normalized and ipsatized (Allik et al., 2015). Previous analyses have shown that, for most personality traits, targets and informants tend to achieve self-other trait agreement in the range of 0.40–0.50 (McCrae et al., 2004; Connolly et al., 2007; Connelly and Ones, 2010). Profile agreements are usually slightly higher than trait agreements (Allik et al., 2015, 2016). Similarly to previous studies, the median profile correlation was *r*_P_ = 0.47 for the Czech sample and *r*_P_ = 0.48 for the Estonian sample. For a profile consisting from 30 facet scores, a statistically significant correlation would be 0.36 (*p* < 0.05). In the Czech sample, 65.5% of dyadic pairs had profile agreements better than this, as did 64.8% in the Estonian sample.

Next, we tried to identify participants in both samples whose low *ICC* scores indicate serious deviations from the typical FFM but who, nevertheless, had a sufficiently high profile agreement *r*_P_. In other words, we looked for deviant or “anomalous” personality profiles, which can be reliably recognized by someone who knows the target sufficiently well.

Unfortunately, there are no set criteria for determining which *ICC* values are low and what level of agreement correlation *r*_P_ is high enough. From a previous study of ours (Allik et al., 2012), we knew that the share of people with atypical personalities in a sample is somewhere in the range from 5 to 10%. To identify such participants, we selected those dyads whose average *ICC* was not statistically significant (*ICC* < 0.26) at *p* < 0.05 but whose self-other agreement was above the sample median correlation, *r*_P_ > 0.47 and *r*_P_ > 0.48, for the Czech and Estonian samples, respectively. As a result, we obtained 63 (7.8%) participants with atypical personality profiles in the Czech sample and 174 (5.2%) participants in the Estonian sample. Although these two criteria (*ICC* < 0.26 and *r*_P_ > 0.47 or *r*_P_ > 0.48) were chosen pragmatically, the reported results changed little when we slightly modified these two cut-off points.

Figure 1A (Czech) and Figure 1B (Estonia) show the mean profiles for participants with a typical FFM and for the minority who has an unusual configuration of personality traits which are, nevertheless, recognizable by those who know this person sufficiently well. It may be surprising that the mean profiles of these two groups are practically identical. Only one subscale out of 30 had a very modest difference in mean values: A4: Compliance for the Czech and O3: Feelings for the Estonian sample (*p* < 0.05). Despite having an almost identical mean level on nearly all subscales, the matrices of covariations only remotely resemble the conventional FFM. This means that “deviants” have on average nearly normal mean levels of traits; what makes them unique is an unusual pattern of correlations between these traits.

Table 2 demonstrates the loadings and congruencies of these factor structures after they were Procrustes rotated toward the American Normative Structure (Costa and McCrae, 1992; Table 5). The overall Tucker's congruence coefficients were 0.84 and 0.80 for the Czech and Estonian deviant groups, respectively. These congruencies indicate that, although the extracted factor structures do have some similarities to the American Normative Structure (Costa and McCrae, 1992; Table 5), they are clearly distinct from it. The five extracted factors explained 56.6 and 44.5% of the total variance for the Czech and Estonian samples, respectively. The fact that mean values remained practically the same in the deviant groups indicates the existence of several heterogeneous subgroups whose mean scores compensate each other.

**Table 2:** Factor loadings for Czech (*N* = 63) and Estonian (*N* = 174) participants whose factor structures deviate from the typical FFM but agree strongly with judgments of acquaintances.

	**Czech sample**						**Estonian sample**					
**Scale**	**N**	**E**	**O**	**A**	**C**	**Congr**	**N**	**E**	**O**	**A**	**C**	**Congr**
N1:Anxiety	**0.84**	0.00	0.17	0.02	0.14	*0.94*	**0.66**	0.09	−0.12	0.03	−0.23	*0.96*
N2:Angry Hostility	**0.64**	−0.03	−0.27	−0.40	0.34	*0.82*	**0.61**	−0.03	−0.23	−0.25	0.14	*0.87*
N3:Depression	**0.84**	−0.10	−0.02	0.09	−0.12	*0.97*	**0.56**	−0.22	−0.06	0.33	−0.23	*0.84*
N4:Self-Consciousness	**0.60**	−**0.56**	−0.07	0.16	−0.06	*0.86*	**0.48**	−**0.40**	−0.10	−0.26	−0.02	*0.80*
N5:Impulsiveness	0.16	**0.66**	0.09	−0.11	−**0.46**	*0.81*	0.37	**0.54**	0.16	−0.35	−0.09	*0.87*
N6:Vulnerability	**0.81**	0.03	−0.14	0.14	−0.05	*0.88*	**0.48**	0.09	−0.22	0.03	−0.40	*0.91*
E1:Warmth	−0.17	**0.65**	0.06	0.32	0.37	*0.94*	−0.23	**0.69**	0.01	0.41	0.06	*0.96*
E2:Gregariousness	−0.05	**0.68**	−0.28	−0.16	−0.08	*0.84*	−0.21	**0.62**	−0.15	0.09	−0.36	*0.87*
E3:Assertiveness	−0.40	0.23	0.16	−**0.47**	0.29	*0.93*	−0.30	**0.46**	0.31	0.02	0.22	*0.87*
E4:Activity	−0.05	**0.42**	**0.58**	−0.19	0.36	*0.84*	−0.08	**0.52**	0.40	−0.23	0.34	*0.93*
E5:Excitement Seeking	0.12	**0.65**	−0.01	−0.17	−0.26	*0.89*	−0.11	0.25	0.17	−0.32	−0.29	*0.79*
E6:Positive Emotions	−0.23	**0.67**	0.15	0.19	0.10	*0.96*	0.13	0.39	0.20	−0.02	0.39	*0.77*
O1:Fantasy	0.34	0.11	**0.51**	0.01	−0.20	*0.93*	0.22	0.27	0.33	−0.44	−0.25	*0.84*
O2:Aesthetics	0.09	−0.08	**0.54**	0.18	0.34	*0.92*	0.24	−0.20	**0.52**	0.24	0.32	*0.87*
O3:Feelings	**0.46**	0.37	0.28	−0.02	**0.49**	*0.85*	0.43	0.28	0.11	−0.03	0.34	*0.79*
O4:Actions	−0.10	0.26	0.41	−0.30	−**0.43**	*0.68*	−0.25	0.05	0.31	−0.36	−0.30	*0.59*
O5:Ideas	−0.27	−0.12	**0.66**	−0.01	0.30	*0.95*	−0.25	−0.07	**0.75**	0.15	0.08	*0.94*
O6:Values	−0.38	0.19	**0.43**	0.08	−0.33	*0.86*	−0.41	0.05	0.04	−**0.45**	−0.42	*0.43*
A1:Trust	−0.01	0.30	**0.50**	0.24	−0.24	*0.57*	−0.46	0.39	0.20	0.17	−0.11	*0.77*
A2:Straightforwardness	0.26	−0.29	−0.27	**0.51**	0.24	*0.85*	0.17	−0.06	0.28	0.30	0.12	*0.60*
A3:Altruism	−0.15	0.43	0.03	**0.50**	**0.50**	*0.94*	−0.15	0.43	−0.26	0.28	−0.09	*0.77*
A4:Compliance	−0.30	−0.22	0.32	**0.54**	0.11	*0.83*	−0.25	−0.21	−0.01	**0.47**	−0.33	*0.80*
A5:Modesty	0.28	−0.18	−**0.41**	**0.46**	−0.13	*0.91*	0.35	−0.41	−0.11	0.32	0.24	*0.68*
A6:Tender-Mindedness	0.16	0.27	−0.00	**0.42**	−0.12	*0.91*	0.34	−0.03	−0.05	0.39	0.43	*0.51*
C1:Competence	−0.36	0.37	0.34	−0.08	**0.54**	*0.92*	−0.57	0.07	−0.02	−0.12	**0.54**	*0.93*
C2:Order	0.66	0.17	−0.09	−0.17	0.20	*0.26*	0.34	−0.03	−0.03	−0.33	**0.43**	*0.62*
C3:Dutifulness	**0.46**	−0.01	−0.33	0.37	**0.51**	*0.55*	0.08	0.17	−0.16	0.08	**0.64**	*0.82*
C4:Achievement Striving	0.06	0.38	0.32	0.02	**0.54**	*0.89*	0.15	0.27	0.28	0.14	**0.59**	*0.86*
C5:Self-Discipline	−0.08	0.04	−0.19	−0.00	**0.65**	*0.93*	−0.11	0.02	−0.28	−0.25	**0.69**	*0.85*
C6:Deliberation	−0.10	−0.28	−0.03	**0.56**	0.23	*0.74*	−0.23	−0.34	−0.36	**0.40**	0.17	*0.70*
*Congruence*	*0.82*	*0.93*	*0.81*	*0.87*	*0.79*	*0.84*	*0.88*	*0.87*	*0.74*	*0.68*	*0.80*	*0.80*

*Congr, Tucker's coefficient of congruence; N, Neuroticism; E, Extraversion; O, Openness to Experience; A, Agreeableness; C, Conscientiousness*.

*Loadings higher than |0.40| are shown in bold, the Tucker's coefficients of congruence are shown in italics*.

These two samples, *N* = 63 and 174, were separately too small for most of the analyses, and even after merging the Czech and Estonian samples, the size of the composite sample (*N* = 237) was still suboptimal for analysis. However, one advantage of the joint sample is its cross-cultural generalizability. We applied different techniques—inverse and hierarchical factor analysis, and K-mean cluster analysis—to identify clusters with similar personality profiles. Similarity between two personality profiles was defined by their Pearson's product moment correlation, which shows the extent to which two profiles have a common shape. The different techniques pointed to four sufficiently distinctive clusters. For example, a hierarchical classification tree of the 237 profiles was possible to be cut into 4 branches which largely overlapped with the classifications made based on the factor analysis and K-means clustering. The inverse factor analysis also identified approximately the same four group of participants with similar personality profiles.

**Figure 2:**
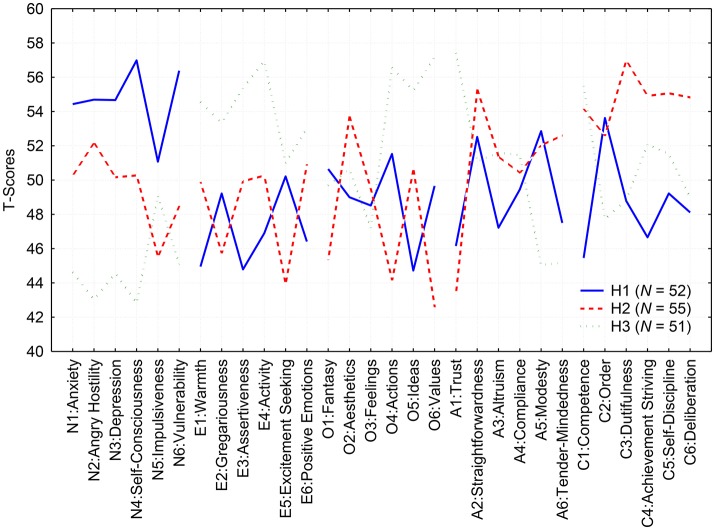
Mean profiles for the three distinctive clusters (H1—solid blue line; H2—dotted red line; H3—fragmentary green line) of participants with deviant personality profiles in the composite sample of Czechs and Estonians.

Next, we computed the mean profiles for these four clusters. We also computed correlations between the individual and mean profiles of these four clusters. We eliminated the fourth cluster because its mean profile barely deflected from the mean value of the standardized *T-*scores. Together with the 4th cluster, we eliminated 79 participants who had lower than *r* = 0.27 (which corresponds to a significance level of about 0.15) correlation with any of the first three mean profiles. After elimination profiles that were unrelated to any of these groups, we recomputed the mean profiles based on those individuals who remained in the first three clusters. Figure 2 demonstrates the mean profiles for these three clusters, labeled as H1, H2, and H3.

*Cluster H1* (*N*_H1_ = 52; 21 Czechs and 31 Estonians). When looking for domains where there are some subscales above the mean 50-point line but others below it, it is obvious that individuals in the first cluster are characterized by a slightly elevated A2: Straightforwardness and A5: Modesty, but with scores below the mean level on the remaining three subscales: A1: Trust, A3: Altruism, and A6: Tendermindedness. Another disparity characterizes Conscientiousness. Individuals in this cluster have a somewhat lower level of Conscientiousness, except for a higher than average level need for C2: Order.

*Cluster H2* (*N*_H2_ = 55; 11 Czechs 11 and 44 Estonians). This cluster is more typical of Estonians than Czechs. The members of this cluster generally have elevated scores on Conscientiousness and a polarization on the subscales of Openness and Agreeableness. They have above average scores on O2: Aesthetics but, at the same time, they are low on scores for Openness for O4: Actions and O6: Values. They are generally frank and sincere with other people (A2: Straightforwardness), but they remain skeptical and are inclined to assume that others may be dishonest (A1: Trust).

*Cluster H3* (*N*_H3_ = 51; 18 Czechs and 33 Estonians). In this cluster, the polarization of subscales involves three different factors. The most distinctive feature of this cluster is a split between the subscales of Openness: members of this cluster have low scores on the first three subscales, O1: Fantasy, O2: Aesthetics, and O3: Feelings, but above average openness to O4: Action, O5: Ideas, and O6: Values. Members of this cluster have a high level of A1: Trust, but, at the same time, they are quite low in A5: Modesty and A6: Straightforwardness. They also have a high level of C1: Competence, but they are not very good at keeping things in C2: Order.

## Discussion

This study demonstrates that besides a usual or dominant personality traits configuration, there are infrequent configurations which clearly deviate from the norm. We are not disputing whether the FFM constitutes an accurate model for the description of human personality. We believe that, in most cases, the customary configuration of the FFM summarizes how most people on this planet describe their own or somebody else's personality, that is, enduring tendencies to think, feel, and behave in a characteristic manner. Although the FFM is dominant in most human cultures where it has been administered so far, it is still only a near-universal, that is, it does not match every individual in every culture. We concluded that the customary FFM configuration is not the only one that exists. We propose that, in addition to the most common personality structure or FFM, there are also rare configurations composed of known personality traits. These rare configurations—unusual patterns of covariations—are not due to response biases or respondents' fantasies; external observers can also see these unusual configurations of personality traits and independently confirm their existence. How do we know that these are not just blunders of personality judgment? Because, as already mentioned, there is outstanding agreement between self-ratings and the ratings of someone who knew this target sufficiently well. Although these variants are infrequent in populations, they may play a significant role in people's lives.

As in anatomical variation among humans, the most common combination of personality traits is probably the best functioning under most circumstances (Leroi, 2005, p. 17). Nevertheless, there are unknown factors responsible for the formation of unusual combination of personality traits. As a comparison, although it may be tempting to believe that all humans have five fingers, surprisingly many people—one in about 3,000—are born with an extra finger or toe (or both) (Leroi, 2005, p. 122). Thus, the most common variant including personality can be defined as “normal” only in the statistical sense. Humans differ from each other in very many ways and it is possible that people differ from one another not only by their makeup in all possible personality facets, but also in the way in which these traits are coalesced into factors that are more general. Usually people who are, for example, frank and sincere with other people are also disposed to trusting others, even if they are not members of their nuclear family. However, we still infrequently find individuals who predominantly behave frankly and openly with other people but at the same time remain reserved in their trusting of others. Thus, these two traits—straightforwardness and trust—which usually go hand in hand, are disassociated in some cases (see cluster H1).

The discovery of rare personality variants—“personality mutants”—contributes to the old debate over whether the FFM reflects actually existing behavioral dispositions or is somehow derived from the meanings of words, without taking into account any actual behavior (D'Andrade, 1965). Of course, it is possible that some people will have somewhat deviant structures in their understanding word meanings—which word meaning goes hand in hand with other word meanings—but it is very unlikely that two persons have the same rare semantic deviations simultaneously. Thus, the existence of personality deviants who are perceived so from the self and the observer perspective makes D'Andrade's words meaning hypothesis not very plausible.

Identification of these rare personality configurations, as we explained above, had been impossible without a reasonable solution to person-fit problems. There was no generally accepted method for evaluating the person-fit for multidimensional latent trait models of personality. Most person-fit statistics used in testing mental abilities are inadequate for this purpose, because they regard the general response pattern of a group as a norm and deviations from this are penalized (Meijer and Sijtsma, 2001; Karabatsos, 2003; Albers et al., 2016). In personality, however, all levels of traits—low, average, and high—are permissible, and none of these can be chosen as a normative reference for all others. Only after developing an acceptable method—computing *ICC*—for evaluating the person-fit to the FFM (Allik et al., 2012) did it become possible to evaluate the universality of FFM at the level of individuals. In this process, it was discovered that there are rare and unusual personality trait configurations, which are, nevertheless, recognizable by external observers.

Despite this first attempt at describing rare personality types, we still know very little about them. Establishing the standard FFM was a long, and even painful, process (John et al., 2008). It is likely that identification of rare types of personality, which may characterize only a few percent of the population, will be process that is even more intricate. Preliminary examinations did not reveal conspicuous associations between these deviants and demographic variables such as age, sex, and education. It may take some time, and considerable effort, to compose a catalog of all personality “deviants” and their consequential outcomes (Ozer and Benet-Martinez, 2006).

There is an analogy with genetics: we probably know more about the most common genetic makeup than we know about rare genetic variations (Zwick et al., 2000), and, more importantly, such rare variations could cause spurious correlations, which are difficult to interpret (Dickson et al., 2010). This is one reason why rare personality trait configurations need to be studied: why they occur and how they persist and function. It may take some time and considerable efforts to compose a catalog of all personality “mutants” and their consequential outcomes (cf.; Ozer and Benet-Martinez, 2006).

One conclusion, however, seems to be inevitable. If we acknowledge the existence of a relatively infrequent group of individuals who have a rare combination of personality facets, then we also need to accept that the FFM is not the only possible template according to which personality facets coalesce into higher order factors. Personality facets are more like Lego, which can be assembled into different configurations. In addition to the customary assembly of elements, several infrequent compositions of the same elements can emerge. The existence of alternatives in the assembling of personality facets seems to validate the network approach (Cramer et al., 2012). The main idea of this approach, as we explained above, is that, instead of there being abstract pre-existing general personality factors, personality structure is formed by learning through networks of mutual dependencies. One event leads to another, which, as a result, modifies the matrix of possible covariations (Cramer et al., 2012). However, it is not entirely clear that the network approach provides a genuine alternative to the Big Five (Wright, 2017). One of the main reasons is that supporters of the FFM were busy with other problems and neglected the question of how the different personality facets coalesce into the five independent factors. The question of which subscales represent the Big Five in an optimal way was usually solved as a pragmatic, and not a theoretical, question (Costa and McCrae, 2017). In the result, a uniform grouping of subtraits was largely assumed, not empirically demonstrated.

The existence of rare personality configurations poses several additional problems but also promises to solve some existing puzzles. The first of these is the pleiotropy of personality indicators, if we may loan this concept from genetics. Compilers of personality questionnaires often have trouble finding “pure” indicators. For example, a tendency to experience anger and related emotions, such as frustration and bitterness, is a good indicator of negative emotions, or Neuroticism in general. However, the N2: Angry Hostility subscale also has the propensity to have a substantial negative loading on a “wrong” factor: Agreeableness. Indeed, a person who is sympathetic to others and eager to help them is not expected to express angry hostility toward others very often. Another example is C1: Competence, with its tendency to load negatively on Neuroticism, besides being a good indicator of Conscientiousness. Competence refers to one's sense that she or he is capable, sensible, and prepared to deal with life's events (Costa and McCrae, 1992). Neuroticism, on the other hand, is the lack of emotional stability and maladjustment to one's life. Thus, there are several pleiotropic NEO PI-R/3 facets that have the tendency to indicate various personality factors simultaneously. Unexpected cross-loadings in the FFM may be caused by minority deviant groups who have unusual combination of personality facets.

The second problem brought about by rare personality configurations is homogeneity of indicators. For example, as the name suggests, enjoying other people's company is a defining feature of Extraversion. However, positive emotions, not sociability, appear to be the central core of Extraversion. Although sociability is undoubtedly an important part of Extraversion—socializing is one of the best source of positive emotions—, extraverts' sociability may be a by-product of the positive emotions received from interacting with other people (Lucas et al., 2000). Analogously, not all Neuroticism subscales correspond equally well to Costa and McCrae's basic definition of this trait (Endler et al., 1997). Because negative affectivity appears to be a central theme binding Neuroticism facets together (Lucas et al., 2000; Markon et al., 2005), it can be expected that N5: Impulsiveness is more peripheral and less strongly connected with the other subscales of Neuroticism. Genetic evidence also supports the idea that different facets may not have an identical genetic background (Realo et al., 2017). There are also several different forms of impulsive behavior, which, due to confusion, can cause spurious correlations on the “wrong” factors (Sharma et al., 2014). Personality disorders may also reveal disassociations between facets. For example, it is common that symptoms of disorders are correlated, not with all subscales of one trait, but selectively with only some of them. As an example, the symptoms of schizotypy—magical ideation and perceptual aberrations—are strongly correlated with the first three subscales of the NEO PI-R Openness subscales, but not much with the rest of them (Ross et al., 2002). Again, it is logical to expect that facets that do not form the core of one of the Big Five factors will have a tendency to leave the intended factor and form relations with facets that belong to other factors (see cluster H2).

Finally, we need to mention a connection between the results reported in this study and the typological approach in general. For example, Jack Block developed the Q-sort technique to define personality types. Individual response profiles derived from Q-sorting were classified according to their closeness to several prototypic profiles (Block, 1971). Many studies have demonstrated that three relatively stable and replicable personality types—Resilient, Overcontrolled, and Underconrolled—can be found (Block and Block, 1980). Although the categorical (type) approach does not often demonstrate superiority over the dimensional approach (Costa et al., 2002; Asendorpf, 2003), we are free to ask how these three personality types—Resilient, Overcontrolled, and Underconrolled—are related to the rare personality types we described above. It is important to note that, in this and previous studies, the majority of participants could be classified into these personality categories or types. These types do not go outside of or beyond the conventional Big Five because the best way to describe them is to specify the personality dimensions on which they score low, average, or high. From the perspective of this study, all three types of personality identified by Block are dominant forms of the standard FFM. However, in this study, we are talking about infrequent personality types that deviate from the standard to which most of us belong.

## Ethic statement

The data collection in Estonia was approved by the Research Ethics Committee of the University of Tartu (approvals: 236/M-29, 14 May 2014; 206/T-4, 22 Aug 2011; 170/T-38, 28 April 2008; 166/T-21, 17 Dec 2007).

## Author contributions

JA was responsible for study conceptualization, data analysis, and report writing; MH and AR were responsible for data collection, data preparation and report writing.

### Conflict of interest statement

The authors declare that the research was conducted in the absence of any commercial or financial relationships that could be construed as a potential conflict of interest.
